# Intrapericardial gossypiboma found 14 years after coronary artery bypass grafting

**DOI:** 10.1186/s13019-019-0889-8

**Published:** 2019-04-08

**Authors:** Fatmir Caushi, Lindita Çoku, Ilir Skenduli, Daniela Xhemalaj, Arian Mezini, Emira Hysa, Francesco Rulli

**Affiliations:** 1 Department of Thoracic Surgery, University Hospital “Shefqet Ndroqi”, Street “Shefqet Ndroqi”, 1001 Tirana, Albania; 2 Department of Cardio-Thoracic Surgery, Mount Sinai Hospital, New York, NY USA; 3 Department of Surgery, “Our Lady of Good Counsel” University, Tirana, Albania

**Keywords:** Gossypiboma, Intrapericardial, Surgery

## Abstract

**Background:**

Foreign body left after surgery surrounded by a foreign body reaction otherwise known as gossypiboma, have been first described in 1884. Although it occurs rarely, it can lead to various complications which include adhesions, abscess formation and related complications. Intrathoracic gossypiboma is a rare but serious consequence of negligence, mainly during abdominal and cardiothoracic surgery that can lead to severe medical consequences. This paper aims to raise awareness among surgeons and nurses in the operating room to prevent such errors and future complications.

**Case presentation:**

A patient with a history of coronary arterial bypass grafting performed 14 years ago, presented with shortness of breath and dry cough. A chest X-ray revealed a large mass in the left hemithorax. The chest CT demonstrated the presence of a heterogeneous density mass of 11 cm and smooth edges in the middle mediastinum, next to the heart and partially intrapericardial. Because clinical and radiologic evidence revealed presence of a mass, we did proceed with CT guided FNA of the mass. The cytology findings confirmed an inflammatory lesion. Based on patient symptomatology and the evidence of a mass, allegedly compressing the cardiopulmonary structures in vicinity, we performed surgical exploration. An old and degraded piece of surgical swap was found and removed through an anterolateral left thoracotomy. The post-operative course was excellent.

**Conclusions:**

Forgetting surgical swaps during surgery is a medical fault. To avoid them, surgical units should design and implement a surgical inventory process to account for surgical instruments or surgical swaps. Failure to make a proper diagnosis of cases such as these can lead to further health complications in these patients. The iatrogenic foreign material seen as a mass in the radiologic films had not been previously noticed by other health professionals although the patient had undergone X-ray and cardiac ultrasound examinations in the 14 years following coronary bypass surgery. Once the causative agent was identified and removed the patient returned to normal activity.

## Background

Gossypiboma (aka Textiloma), described first in 1884, is a mass within the body composed of a cotton matrix, which usually refers to a retained surgical sponge or swap, surrounded by a foreign body reaction [1]. Although it occurs rarely (1/1000–1500 abdominal operations), it has significant associated morbidity and mortality. Pathologically, the body response to the foreign body occurs either as an aseptic fibrosis response, or as an exudative reaction resulting in localized abscess formation [2]. Intrathoracic gossypiboma is a rare but serious consequence of negligence, mainly during abdominal and cardiothoracic surgery that can have severe medical consequences [1]. The patient with intrathoracic gossypiboma may present symptoms like fever, cough, hemoptysis, weight loss, dyspnea, and shoulder pain [3].

## Case presentation

**Fig. 1:**
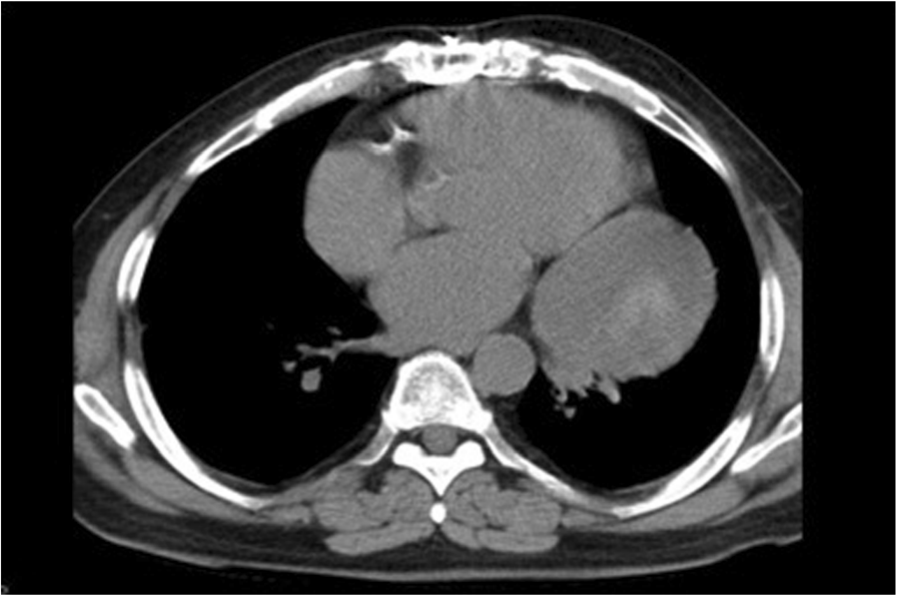
The Chest CT demonstrates a mass with smooth edges and heterogeneous density, next to the heart and partially intrapericardial

A 50 year-old male, with a history of coronary arterial bypass grafting 14 years back, presented with shortness of breath and dry cough. An X-ray revealed a large mass in the left hemithorax adjacent to the heart silhouette. A chest CT demonstrated the presence of a mass with smooth edges, in middle mediastinum next to the heart and partially intrapericardial (Fig. 1). The mass was of heterogeneous density and of 11 cm size. Presence of atelectasis at the left lower lobe abating the mass was clearly seen. Based on clinical and radiologic evidence, we did proceed with CT guided FNA of the mass. The cytology findings revealed inflammatory lesion. Laboratory tests were normal. Based on patient symptoms, history and the presence of a mass potentially compressing the cardiopulmonary structures in vicinity, we decided to offer exploratory surgery for diagnosis and treatment.

**Fig. 2:**
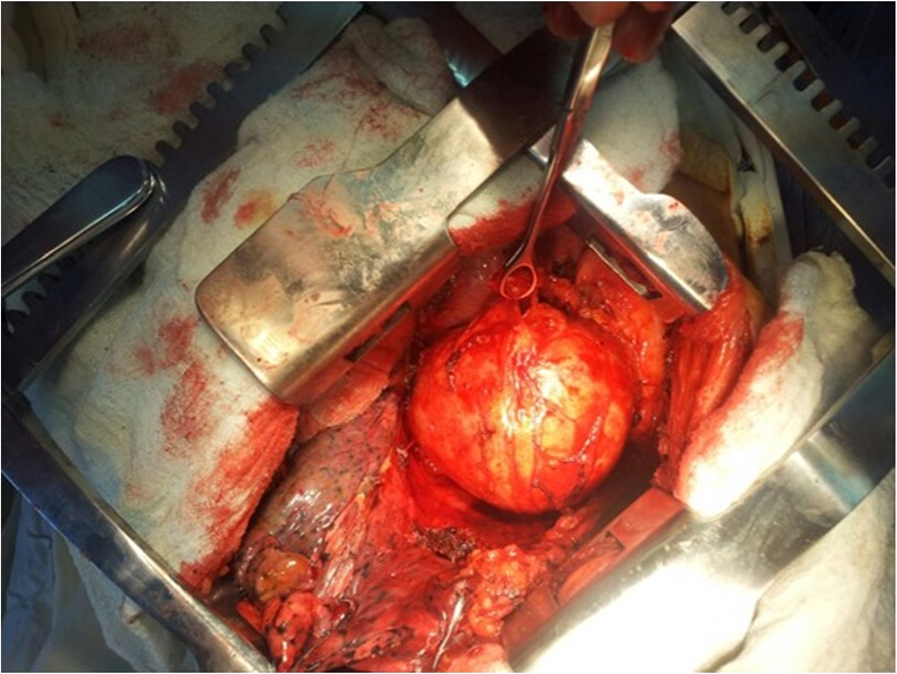
The view of the mediastinal mass after the left thoracotomy

**Fig. 3:**
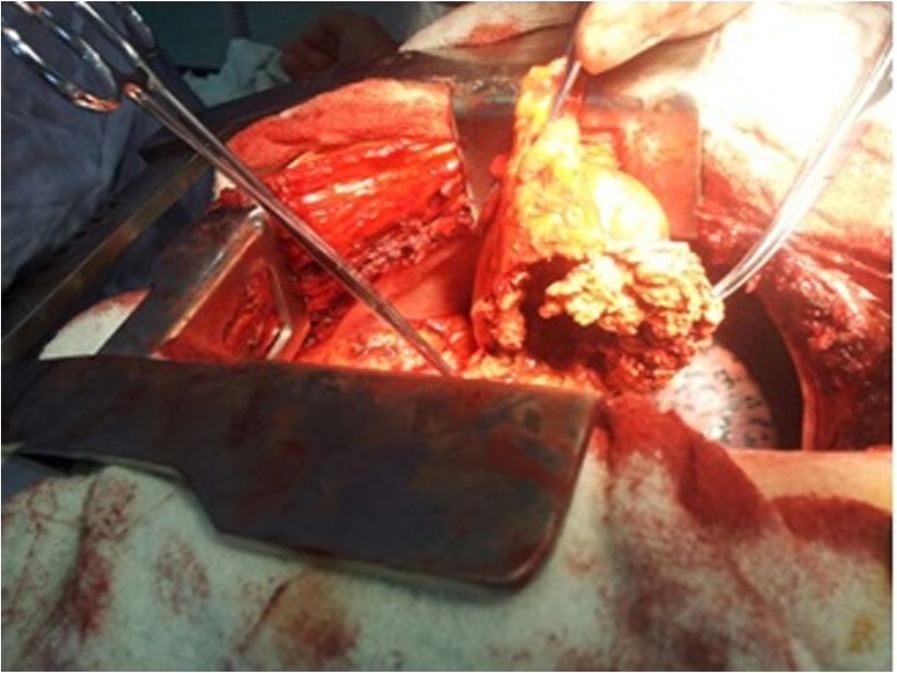
The presence of an old and degraded piece of surgical swap intrapericardial

Standard hemodynamic monitoring and general anesthesia were followed by positioning, prepping and draping patient in left lateral decubitus position. An anterolateral left thoracotomy was carried out and entrance in the hemithorax was made without any challenge. The mass was assessed and found to be leaning medially on the surface of the lateral wall of the left ventricle, including the pericardial layer and had smooth edges which didn’t infiltrate the lung (Fig. 2). We started dissecting the mass from its smooth capsule, making it through all its layers. An old and degraded piece of surgical swap was visualized (Fig. 3). The surgical swap was removed along with the capsular layer of this mass. Patient tolerated the procedure very well and blood loss was minimal. A chest tube was inserted in the left hemithorax and chest wall was closed following standard procedures.

In the immediate post-operative phase, patient improved steadily and on day four was discharged home symptom-free. In the long-term follow -up, patient was found to remain without symptoms.

## Discussion

Gossypiboma is an important topic and perhaps under reported due to their medicolegal implications [4]. Intrathoracic gossypiboma mainly occurs in pleural and pericardial cavities, with only a few cases of intrapulmonary gossypiboma published [3]. Gossypiboma occurs as a result of extensive wound explorations, errors made during counting, lack of using radio-opaque sponges and misreading postoperative radiologic X-ray graphics [2].

A systematic literature review of 254 gossypiboma case studies, identified via the National Library of Medicine’s MEDLINE and the Cochrane Library, revealed that gossypibomas happen most commonly in the abdomen (56%), pelvis (18%) and thorax (11%). Longest reported cases of gossypiboma were found in thorax (43 and 44 years) [5]. There is no clear incidence reported in the literature. An estimated incidence is 1/1000–1/10000 surgeries. The average time to diagnose gossypiboma was 6.9 years and 38% of the cases are identified within the first year from the surgery [2, 4, 6].

CT scanning is the most helpful procedure in diagnosing a gossypiboma, especially if a radiopaque marker is used [4]. Wan et al. found that CT was helpful in the diagnosis of 61% of the cases reviewed, followed by radiography (35%) and ultrasound (4%). Pain/irritation (42%), palpable mass (27%), and fever (12%) were the leading signs and symptoms while 6% of cases were asymptomatic. Common complications of gossypiboma included adhesion (31%), abscess formation (24%), and fistula (20%) [7].

The CT findings change according to type of foreign material, their anatomical location, and type of reactions they generate in the host [1]. .In the case of a surgical swap, the CT scan typically shows a well-encapsulated mass, usually of low density, with calcified deposits in between the fragments of retained surgical swap, exhibiting the ‘calcified reticulate rind’ sign, reported previously. Gossypiboma gives a spongiform appearance with gas bubbles, except when it is located in the thorax, when air is resorbed by the surrounding pleura so no gas opacities are seen [2]. In the early postoperative period, the differential diagnosis of gossypiboma includes hematoma and abscess [3]. Following contrast administration, peripheral rim enhancement can be observed [1].

.MRI and biopsy can also be helpful. However, MRI features can be confusing, while biopsy is often indecisive [4, 8].

Although rare nowadays, gossypiboma cases do occur. It is clear that leaving unintendedly surgical swaps inside the body during an intervention is a medical liability. Efforts should be made to avoid such iatrogenic errors. Implementing OR checklist pre and post surgery, portable ontable detective rings and strict instrument and sponge count is paramount to prevent such potential catastrophes for patients.

In our case, the radiologic image was initially showing a big mass of mediastinum that involved the pericardium and was accompanied by shortness of breath and dry cough. The history of past surgery in this patient additionally suggested that an intrapericardial foreign body could be producing the current symptoms. We decided to perform the exploratory surgery as the best diagnostic and treatment option because it appeared very well circumscribed.

Although this error didn’t happen to us, since this case, we have been more cautious in our surgical practice, counting everything at the beginning of the surgery and before chest closure as described by WHO checklist. We don’t close the thorax if something is missing and some time we have used the C-arm to control. We are currently using only surgical swap with radio opaque marker inside.

## Conclusions

Forgetting surgical swaps inside the body during surgery is a preventable medical error that should be avoid at all cost, even in very difficult cases with a high level of tension in the operating room. They can be prevented by implementing a strict patient safety protocol such as an instrument, needle and surgical swaps count procedure before surgery and before closure. Another lesson learned is that in cases such as ours, surgical exploration is the best treatment. Investigating duration of symptoms, the difficulty to make the correct diagnosis by only routine clinical exam prompts us as surgeons to carefully investigate and intervene in timely fashion. As demonstrated, the iatrogenic foreign material seen as a mass in the radiologic films was the culprit of patient’s severe symptoms. Once the cause was identified and the foreign body removed, patient returned to normal activity.

It is inexcusable that this patient for 14 years made a lot of medical tests, including yearly X-rays and cardiac ultrasounds and no one has found anything wrong. This case aims to raise awareness among surgeons and nurses in the operating room and doctors in their everyday work to prevent such errors and future complications that may deteriorate patients’ health.

## Abbreviations

CT: Computed Tomography
FNA: Fine Needle Aspiration
MRI: Magnetic Resonance Imaging
OR: Operation Room
